# Supplementary material for: Asymmetry Thresholds Reflecting the Visual Assessment of Forelimb Lameness on Circles on a Hard Surface
Source: Animals (Basel). 2023 Oct 25;13(21):3319. doi: 10.3390/ani13213319 (PMC10650068; doi:10.3390/ani13213319)

Figure S1: Flowchart of the inclusion criteria of the screened horses, which were visually considered as single forelimb lame at lunge on both reins on a hard surface by an expert veterinarian during his routine practice.

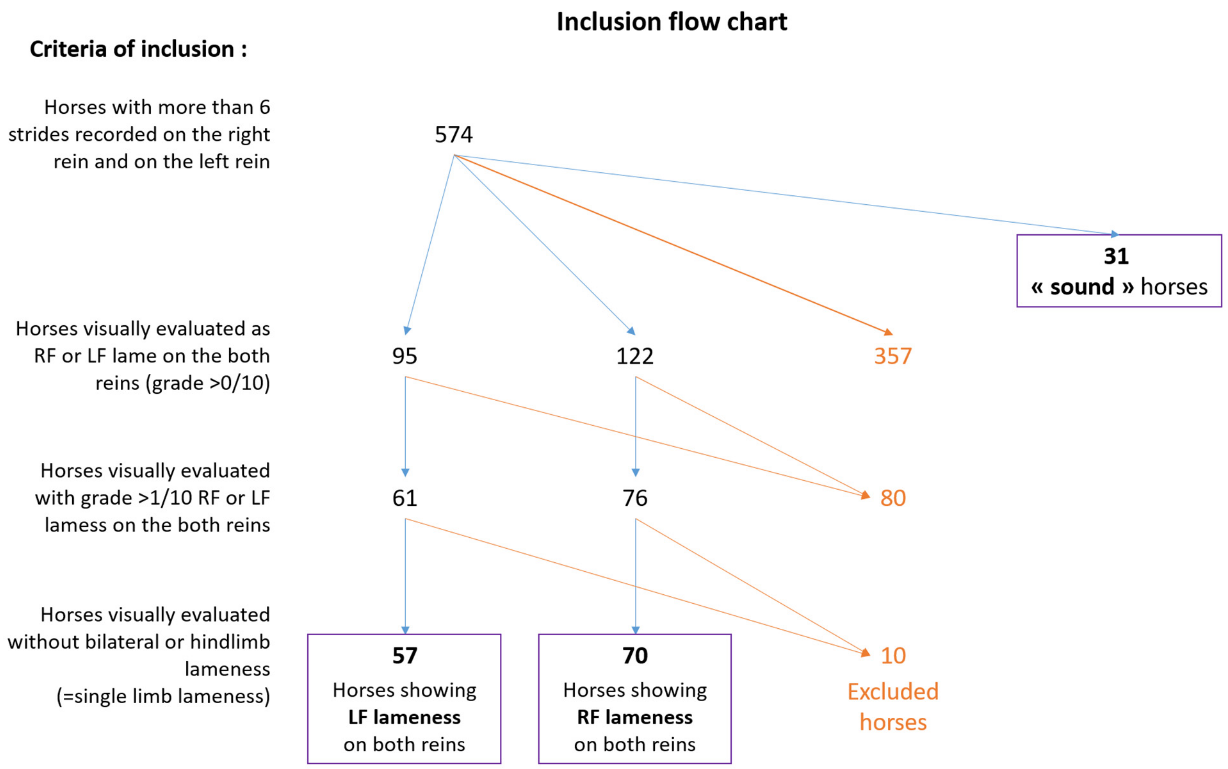

Supplement: Supplementary file 1 [file animals-13-03319-s001.zip › Figure S1.pdf]
